# On Target Localization Using Combined RSS and AoA Measurements

**DOI:** 10.3390/s18041266

**Published:** 2018-04-19

**Authors:** Slavisa Tomic, Marko Beko, Rui Dinis, Luís Bernardo

**Affiliations:** 1 COPELABS, Universidade Lusófona de Humanidades e Tecnologias, Campo Grande 376, 1749-024 Lisboa, Portugal; beko.marko@ulusofona.pt; 2 ISR/IST, LARSyS, Universidade de Lisboa, Av. Rovisco Pais 1, 1049-001 Lisbon, Portugal; 3 CTS/UNINOVA, Campus da FCT/UNL, Monte de Caparica, 2829-516 Caparica, Portugal; 4 Dep.^o^ de Eng.^a^ Electrotécnica, Faculdade de Ciências e Tecnologia, FCT, Universidade Nova de Lisboa, 2829-516 Caparica, Portugal; rdinis@fct.unl.pt (R.D.); lflb@fct.unl.pt (L.B.); 5 Instituto de Telecomunicações, Av. Rovisco Pais 1, Torre Norte, piso 10, 1049-001 Lisboa, Portugal

**Keywords:** target localization and tracking, wireless sensor network (WSN), hybrid measurements, received signal strength (RSS), angle of arrival (AoA)

## Abstract

This work revises existing solutions for a problem of target localization in wireless sensor networks (WSNs), utilizing integrated measurements, namely received signal strength (RSS) and angle of arrival (AoA). The problem of RSS/AoA-based target localization became very popular in the research community recently, owing to its great applicability potential and relatively low implementation cost. Therefore, here, a comprehensive study of the state-of-the-art (SoA) solutions and their detailed analysis is presented. The beginning of this work starts by considering the SoA approaches based on convex relaxation techniques (more computationally complex in general), and it goes through other (less computationally complex) approaches, as well, such as the ones based on the generalized trust region sub-problems framework and linear least squares. Furthermore, a detailed analysis of the computational complexity of each solution is reviewed. Furthermore, an extensive set of simulation results is presented. Finally, the main conclusions are summarized, and a set of future aspects and trends that might be interesting for future research in this area is identified.

## 1. Introduction

### 1.1. Motivation

**Figure 1:**
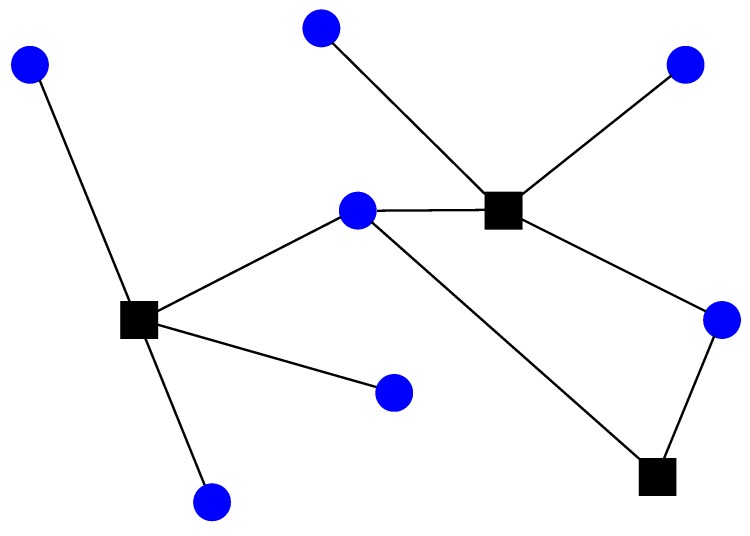
Example of a WSN with three anchors (denoted by black squares) and seven targets (denoted by blue circles).

**Figure 2:**
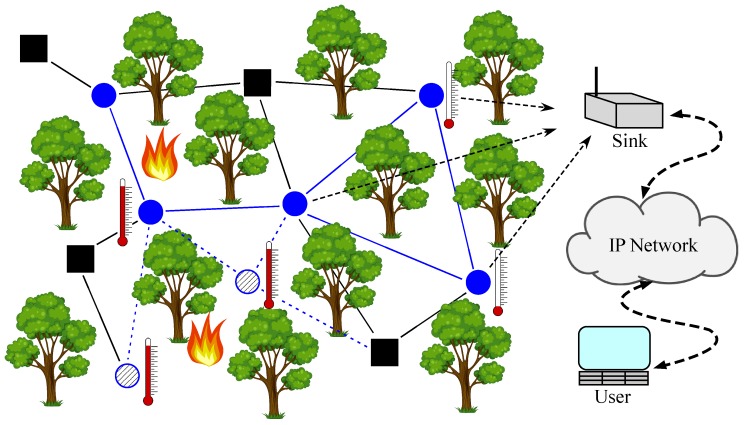
Application of a WSN in forest fire detection/prevention.

The wireless sensor network (WSN) is a wireless communication network consisting of sensor nodes, usually distributed over an area of interest with the goal of measuring a certain quantity (e.g., temperature, pressure, wind, etc.) [1]. Generally, two types of sensors can be distinguished: anchors (their locations are known a priori, e.g., manually positioned by a network administrator) and targets (sensors whose location are to be determined); see Figure 1. Owing to advancements in radio frequency (RF) and micro-electro-mechanical systems, large-scale networks composed of a vast number of sensor nodes (hundreds or even thousands of nodes) are in use nowadays [1]. Because they are autonomous in terms of human interaction and sensor nodes are relatively inexpensive, the application potential of WSNs is huge. They are used in many diverse areas, such as monitoring (healthcare, industrial, environmental, agricultural) [2,3], event detection (floods, hailstorms, fires) [4], exploration (outer space, deep water, underground) [5], surveillance [6] and energy-efficient routing [7], to name a few. A possible application in fire detection/prevention in forests is illustrated in Figure 2. In the figure, some targets might be dropped out of an airplane or they can be mobile. They are used to measure the temperature in their vicinity. As soon as one of them detects a high temperature (fire danger), they can communicate their location, together with a valid warning message to the user (fire-fighters) through a sink.

In many practical applications (such as search and rescue, target tracking and detection, cooperative sensing and many more), data acquired by sensor nodes in a WSN are only relevant if they are linked with the locations of sensor nodes [8,9,10,11,12,13,14,15]. Moreover, many new applications in both emergency and commercial services can be enabled if precise localization of people and objects in both indoor and outdoor environments can be achieved (e.g., location-aware vehicles [16], asset management in warehouses [17], navigation [18,19,20,21], etc.). Since any device in the network can provide a faster and better response to the changes in its surroundings, these applications could improve efficiency and safety in our daily lives [22]. Another area with rapidly growing interest where accurate location of people and/or objects might play a very important role is ambient assisted living (AAL) [23] or smart indoor positioning for situation awareness in emergency situations [24]. In these applications, localization can be useful to identify patterns of movements and trigger alerts when anomalies are detected or to assist fire-fighters returning to a safe zone from dense smoke environments. Therefore, being able to accurately determine the locations of sensor nodes provides additional understanding of the changes in the area of interest to the user and is therefore a valuable resource.

However, sensor nodes are small devices, with low cost and limited power (battery power). Typically, they are distributed in large number across an area with limited, or even no, control of their locations in space (for instance, they can be released out of a chopper for sensing in adverse environments [25]). Besides sensing, sensors have a limited capability of communicating and processing the acquired data (due to their battery life). Installing a global navigation satellite system (GNSS) receiver in each sensor is a possible solution, but it would intensely elevate the network cost, thus limiting its applicability potential [26]. Besides, GNSS is ineffective in indoor, dense urban and forest environments or canyons [27]. With the objective of preserving low implementation costs, only a small portion of sensor nodes are equipped with GNSS receivers or manually positioned by a network administrator, whilst the rest of the sensors establish their locations resorting to an alternative localization scheme that exploits the known locations of the anchors (reference points) [28]. Because sensor nodes have very limited processing capacities, the key requirement for localization schemes is that they are fast, scalable and modest computational and communication requirements. Furthermore, making use of already deployed technologies (like terrestrial RF sources) when determining object’s location is highly appreciated. Nevertheless, WSNs are prone to changes in topology (for instance, due to sensor mobility, sensor addition, sensor and/or link failure), which exacerbates the design of even the simplest schemes.

The concept of wireless positioning was initially devised for cellular networks, because it enables plenty of innovative services and applications for its users. Today, the large spread of heterogeneous smart-devices (cell phones, tablets, etc.) that provide self-sustained applications and seamless access to different wireless networks is making location information an essential part of mobile context-aware applications [28]. Even though the discussion is limited to sensor localization in WSN here, it is worth noting that, in practice, a base station or an access point in a local area network (LAN) can be considered as an anchor, while other devices such as cell phones, laptops, tags, etc., can be considered as targets.

Nowadays, terrestrial RF signals come from a wide variety of sources and technologies, and they can be used for localization purposes. Location information can be acquired through range-based or range-free observations. Here, the focus is on the former ones exclusively, since in general, a higher estimation accuracy can be achieved. Hence, the locations of the targets in a WSN are determined by using an alternative localization scheme that relies on the known locations of the anchors and range measurements between targets and anchors. Range (distance) estimation can be obtained from different properties of the received radio signal, like time of arrival (ToA) [29], time-difference of arrival (TDoA) [30], round-trip time (RTT), time of flight (ToF) [31], angle of arrival (AoA) [32] or received signal strength (RSS) [33,34], to name a few. Which one will be used commonly depends on the available hardware in the sensor nodes. The trade-off between the localization accuracy and the implementation complexity of any method is a relevant factor when choosing which technique to employ. For instance, localization schemes based on ToA or TDoA (including GNSS) offer high precision, but this comes at the cost of a very complex process of timing and synchronization [35]. Hence, the localization process becomes very costly. Even though less precise than systems employing ToA, TDoA or AoA information in general, localization systems based on RSS observations need no specialized hardware, their computational and communication burden is significantly lighter (and thus, they are very energy efficient). Consequently, these systems represent an attractive low-cost solution for the localization problem [1,26]. Besides RSS, localization techniques based on RTT observations are also attractive and low cost. These observations are easily acquired in wireless LAN (WLAN) systems by exploiting a simple device such as a printed circuit board [36]. Although RTT systems do not have the problem of clock synchronization between sensors, their major setback is that they require double signal transmission to acquire just a single measurement [37].

### 1.2. Related Work

The approaches in [38,39,40,41,42,43] studied both the target localization problems in both non-cooperative and cooperative WSNs (the terms non-cooperative and cooperative WSNs are used here to denote the localization problems in which the targets are permitted to exchange information with anchors only or any sensor node within their communication range (whether they are anchors or targets), respectively). Nevertheless, these estimators are all based on RSS and range observations exclusively. The algorithms described in [36,44,45] are founded on the integration of RSS and ToA observations. Another hybrid system that combines range and angle measurements was studied in [46]. Two estimators for the non-cooperative target localization problem in a three-dimensional space were proposed in [46]: linear least squares (LS) and optimization based. The former is a relatively simple and well-known estimator, while the latter was solved by the Davidon–Fletcher–Powell algorithm [47]. In [48], an LS and a maximum likelihood (ML) estimator for a hybrid scheme that merges RSS difference (RSSD) and AoA observations were derived. To estimate the target’s location from multiple RSS and AoA observations, the authors in [48] used a non-linear constrained optimization. However, both LS and ML estimators are λ-dependent, where λ represents a non-negative weight assigned to regulate the contribution from RSS and AoA observations. In [49], the authors described a selective weighted LS (WLS) estimator for the RSS/AoA localization problem. The target location was determined by taking advantage of weighted ranges from the two nearest anchor observations. These were then integrated with the serving base station’s AoA observation. Nonetheless, like [48], in [49], the authors only investigated the non-cooperative hybrid RSS/AoA localization problem in a two-dimensional space. Another WLS approach was proposed in [50]. This estimator was designed for a three-dimensional RSSD/AoA non-cooperative localization problem for unknown transmit power. Even so, the authors in [50] studied a small-scale WSN, with extremely low noise powers only. In [51], the authors presented an estimator founded on the semidefinite programming (SDP) relaxation technique for the cooperative target localization problem. The method in [51] is an extended version of the previous SDP algorithm, developed by the same authors, for pure range information into a hybrid one, by adding angle information for a triplet of points. Owing to the use of triplets of points, the computational complexity of the SDP approach grows significantly with the network size. Two estimators for the three-dimensional RSS/AoA localization problem in non-cooperative WSNs founded on the second-order cone programming (SOCP) relaxation technique and squared-range (SR) approach to convert the localization problem into a generalized trust region sub-problem (GTRS) framework were proposed in [52]. The work in [53] addressed the RSS/AoA non-cooperative localization problem in two-dimensional non-line of sight (NLoS) environments. The authors in [53] proposed an alternating optimization algorithm, composed of fixing the value of the scatter orientation and solving the SDP representation of the localization problem and later using the obtained location estimate to update the value of the scatter orientation, for localizing a mobile target in a WSN. In [14,54], a cooperative RSS/AoA localization problem was investigated. The authors in both [14,54] proposed an SDP estimator to simultaneously localize multiple targets. However, the proposed algorithms are for centralized applications only, and their computational complexity depends highly on the network size. Distributed algorithms based on convex optimization techniques were proposed in [13,15,55] to solve the cooperative RSS/AoA target localization problem with unknown transmit powers in a large-scale WSN. Although the computational burden of the distributed approaches does not depend on the size of the network, but rather on the size of neighborhood fragments, they are executed iteratively, which makes them sensitive to error propagation and increases energy consumption.

## 2. Hybrid RSS/AoA Localization

Range-based localization techniques are widely used nowadays owing to their potentially high accuracy, applicability to different radio technologies and ease of implementation [56]. Within this approach, one can distinguish between range- and range-difference-based methods. Furthermore, another widely-used approach for localization nowadays is based on the angular approach. In the following text, a geometrical interpretation of some well-known range- and angle-based localization techniques is given.

**Figure 3:**
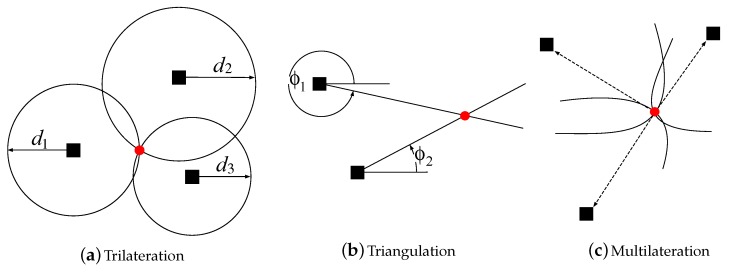
Illustration of geometric-based approaches.

In the case where the noise is absent and the number of anchors is low, geometric-based techniques are appealing, owing to their simplicity. Some basic and intuitive geometric methods are trilateration, triangulation and multilateration; see Figure 3. The trilateration technique makes use of distance measurement and the known location of the anchor to describe a circle around the anchor with the radius equal to the distance measurement [57]. Then, by using at least three anchors in a two-dimensional space, it locates the target by calculating the intersection of the circles based on simultaneous range measurements from the anchors, as shown in Figure 3a. Triangulation is used when the direction of the target instead of the distance is estimated, as shown in Figure 3b. The target location is determined by using the trigonometry laws of sine and cosine [58]. Multilateration is a technique based on the measurement of the difference in distance to two or more anchors that form a hyperbolic curve [59]. The intersection of the hyperbolas, corresponding to the TDoA measurements, determines the position of the target; see Figure 3c. Obviously, at least three hyperbolas are needed to unambiguously determine the unknown target location, which corresponds to at least four anchors.

**Figure 4:**
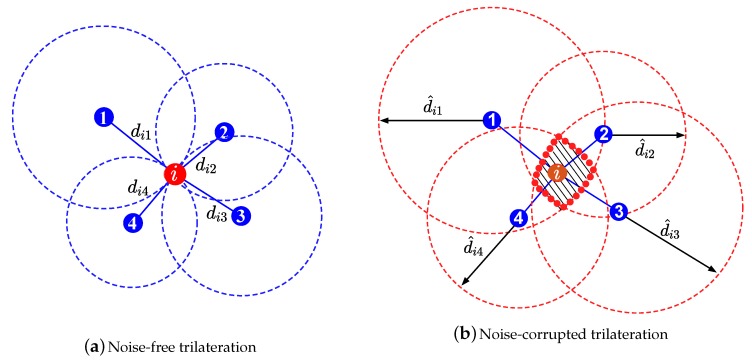
Illustration of a range-based localization principle.

However, in the presence of noise, the intersection of these lines would form an area rather than a single point (see Figure 4), due to noise corruption in the measurements or the increased number of parameters. In such cases, these geometric approaches are not able to provide a useful insight as to which intersection point to choose as the target’s location estimate. Intuitively, the bigger the area formed by the intersections is, the bigger the set of all possible solution will be (more difficult to obtain an accurate estimate of the target’s location).

**Figure 5:**
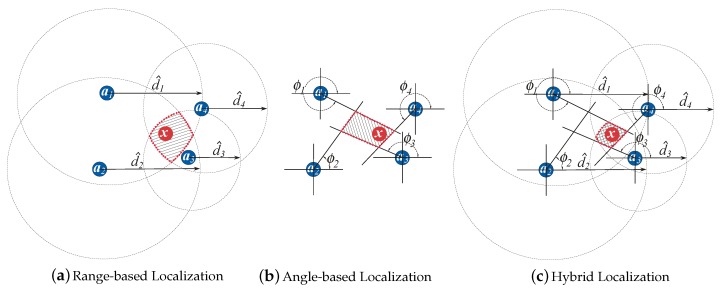
Illustration of different localization systems in a 2D space.

Nevertheless, this area can be reduced significantly if the two measurements (range and angle) are integrated together. Figure 5 depicts how (a) range-based, (b) angle-based and (c) hybrid (range and angle) systems function in a network with M=1 and N=4, in the presence of noise. As before, in the range-based systems, each range observation, ${\hat{d}}_{i}$, is used to form a circle as an area containing the unknown target. Hence, a sequence of range observations, $\left\{{\hat{d}}_{1},{\hat{d}}_{2},\ldots ,{\hat{d}}_{N}\right\}$, forms multiple circles, and the area defined by their intersection accommodates the target, as illustrated in Figure 5a. Similarly, in the case of angle-based systems, each angle observation, $\phi_{i}$, is used to define a line in the direction of the unknown target location, as illustrated in Figure 5b. From Figure 5c, it can be seen that by joining the two observations of the radio signal, the area formed by the intersection, i.e., the set of all possible solutions for the unknown target location, is substantially smaller than in each individual case. Therefore, the use of hybrid systems is more likely to improve the localization accuracy; hence, in this work, we will focus on the hybrid systems exclusively and more precisely on the RSS/AoA systems.

### 2.1. Problem Formulation

**Figure 6:**
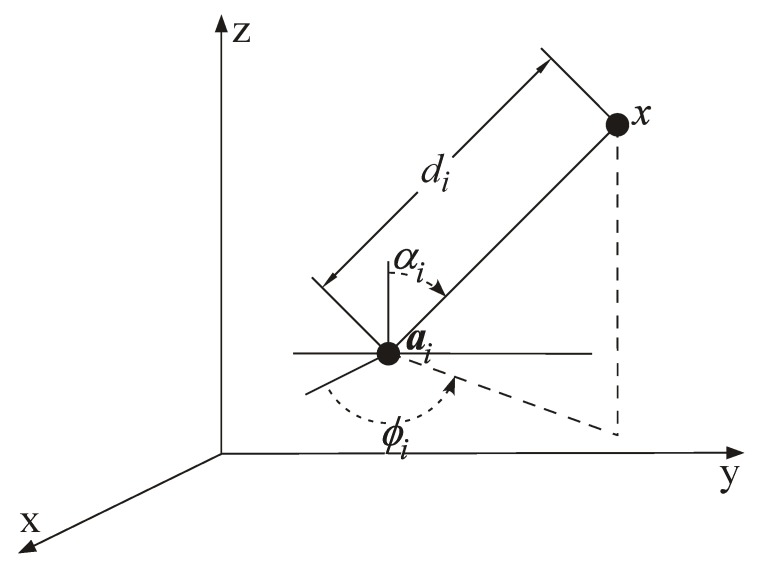
Graphical illustration of the measurement models in a three-dimensional space.

This section introduces the RSS and AoA observation models and formulates the localization problem in a three-dimensional space. Let $x\in R^{q}$ (where q=3) be the unknown location of the target and $a_{i}\in R^{q}$ be the known location of the *i*-th anchor, with i=1,…,N. To estimate the target’s location, a hybrid scheme that integrates range and angle observations is utilized, as shown in Figure 6. In Figure 6, $x={\left[x_{x},x_{y},x_{z}\right]}^{T}$ and $a_{i}={\left[a_{ix},a_{iy},a_{iz}\right]}^{T}$ denote the true coordinates of the target and the *i*-th anchor, respectively, whereas $d_{i}$, $\phi_{i}$ and $\alpha_{i}$ represent respectively the distance, azimuth angle and elevation angle between the target and the *i*-th anchor.

Here, it is assumed that the distance estimates are extracted from the RSS information exclusively. This is mainly because ranging based on RSS requires no additional hardware [26]. The noise-free RSS between the target and the *i*-th anchor is defined as [60,61] (Ch. 3):

(1) $$P_{i}=P_{T}{\left(\frac{d_{0}}{d_{i}}\right)}^{\gamma }10^{-\frac{L_{0}}{10}},\;\;\mathrm{for}\;\;i=1,\ldots ,N,$$

where $P_{T}$ is the transmit power of the target, $L_{0}$ is the path loss value measured at a short reference distance $d_{0}$ ($d_{0}\leq d_{i}$), γ is the path loss exponent (PLE) and $d_{i}$ is the distance between the target and the *i*-th anchor. The RSS model in (1) can also be written in a log-distance form as:

(2) $$P_{i}\;\left(\mathrm{dBm}\right)=P_{0}-10\gamma \log_{10}\frac{d_{i}}{d_{0}}+n_{i},\;\;\mathrm{for}\;\;i=1,\ldots ,N,$$

where $P_{0}\;\left(\mathrm{dBm}\right)=P_{T}\;\left(\mathrm{dBm}\right)-L_{0}\;\left(\mathrm{dB}\right)$ is the RSS received at $d_{0}$ and $n_{i}∼N\left(0,\sigma_{n_{i}}^{2}\right)$ is the log-normal shadowing term modeled as a zero-mean Gaussian random variable with standard deviation $\sigma_{n_{i}}$ (dB). Note that $P_{0}$ is dependent on $P_{T}$.

On the other hand, the AoA observations can be acquired by implementing a directional antenna, or an antenna array [46,62], or even video cameras [63] at anchors. Therefore, by following simple geometry, azimuth and elevation angle observations are modeled as [46]:

(3) $$\phi_{i}=\tan^{-1}\left(\frac{x_{y}-a_{iy}}{x_{x}-a_{ix}}\right)+m_{i},\;\;\mathrm{for}\;\;i=1,\ldots ,N,$$

(4) $$\alpha_{i}=\cos^{-1}\left(\frac{x_{z}-a_{iz}}{∥x-a_{i}∥}\right)+v_{i},\;\;\mathrm{for}\;\;i=1,\ldots ,N,$$

respectively, where $m_{i}\;\left(\mathrm{rad}\right),v_{i}\;\left(\mathrm{rad}\right)$ are the measurement errors of the azimuth and elevation angles, respectively, modeled as $m_{i}∼N\left(0,\sigma_{m_{i}}^{2}\right)$ and $v_{i}∼N\left(0,\sigma_{v_{i}}^{2}\right)$.

By defining the observation vector as $\theta ={\left[P^{T},\phi^{T},\alpha^{T}\right]}^{T}$ ($\theta \in R^{3N}$), where $P=[P_{i}]$, $\phi =[\phi_{i}]$, $\alpha =[\alpha_{i}]$, the conditional probability density function (PDF) can be written as:

(5) $$p\left(\theta |x\right)=\prod_{i=1}^{3N}\frac{1}{\sqrt{2\pi \sigma_{i}^{2}}}\exp\left\{-\frac{{\left(\theta_{i}-f_{i}\left(x\right)\right)}^{2}}{2\sigma_{i}^{2}}\right\},$$

where $\sigma ={\left[\sigma_{n_{i}},\sigma_{m_{i}},\sigma_{v_{i}}\right]}^{T}$ and $f\left(x\right)={\left[P_{0}-10\gamma \log_{10}\frac{d_{i}}{d_{0}},\tan^{-1}\left(\frac{x_{y}-a_{iy}}{x_{x}-a_{ix}}\right),\cos^{-1}\left(\frac{x_{z}-a_{iz}}{∥x-a_{i}∥}\right)\right]}^{T}$.

The ML estimate, $\hat{x}$, of the unknown location can be obtained by maximizing the log of the likelihood function (5) with respect to x [64] (Ch. 7), as:

(6) $$\hat{x}=\underset{x}{\arg\;\min}\sum_{i=1}^{3N}\frac{1}{\sigma_{i}^{2}}{\left[\theta_{i}-f_{i}\left(x\right)\right]}^{2}.$$

**Figure 7:**
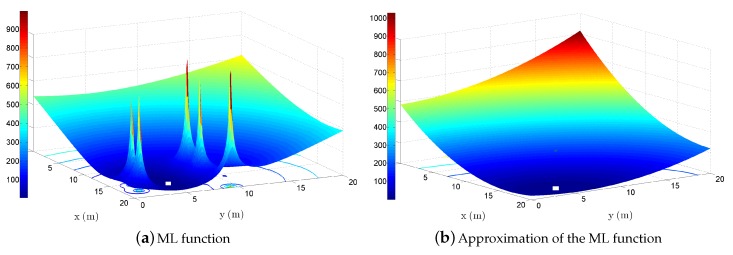
Illustration of the optimization-based principle; the true target location at ${\left[17.35,4.77\right]}^{T}$.

The above ML estimator (6) is very challenging: it is highly non-convex, and its solution cannot be obtained in closed-form. Solving it directly (e.g., by a grid search algorithm) could be very exhaustive computation-wise, and relying on recursive approaches (e.g., gradient descent algorithm) could produce poor localization accuracy due to the non-convexity; see Figure 7a. Therefore, it is shown in the following text that (6) can be tightly approximated by another estimator whose global optima is readily obtained, as illustrated in Figure 7b.

Figure 7 illustrates the main idea when solving the localization problem used in the literature. It represents a possible realization of the objective function in (6), shown in Figure 7a, and a possible realization of an approximation of (6), shown in Figure 7b. Approximation of a non-convex estimator by a convex one is a common approach used by algorithms based on convex optimization, such as the ones presented in Section 2. For the purpose of this illustration, all sensors were randomly placed inside a square region of $20\times 20\;m^{2}$. The true target’s location was set at ${\left[17.35,4.77\right]}^{T}$, and N=5 anchors were able to directly communicate with the target. The rest of the parameters we set as follows: $P_{0}=-10$ dBm, $\sigma_{n_{i}}=5$ dB, $\sigma_{m_{i}}=8$ deg, $\sigma_{v_{i}}=8$ deg, γ=3, and the objective functions were plotted versus *x* (m) and *y* (m) coordinates (the step in the mesh grid was 0.1 m). Figure 7a shows that the objective function in (6), given the true positions of the targets, has a global minimum at ${\left[17.5,4.7\right]}^{T}$ and some local minima and saddle points around it. Figure 7b shows that the approximated objective function has a global minimum at ${\left[18,4.2\right]}^{T}$ and is much smoother than (6). One can also see that the two objective functions have a similar behavior: both monotonically increase and decrease in the same regions. Hence, from Figure 7, it is clear that the objective function in (6) can be tightly approximated by another. Since the global solution of the latter objective function can be readily obtained, the idea of approximating (6) seems a valid choice. Obviously, the quality of the final solution (localization accuracy) will depend on the tightness of the applied approximations.

In the remainder of this section, we will present a short review of a set of existing, state-of-the-art estimators for target localization in a three-dimensional space, using integrated RSS and AoA measurements.

### 2.2. SDP Estimator

In [14], the authors showed how to approximate the non-convex ML problem in (6) by an SDP one. First, from (2), (3) and (4), respectively, it is written:

(7) $$\lambda_{i}∥x-a_{i}∥\approx \eta d_{0}\;\;\mathrm{for}\;\;i=1,\ldots ,N,$$

(8) $$c_{i}^{T}\left(x-a_{i}\right)\approx 0,\;\;\mathrm{for}\;\;i=1,\ldots ,N,$$

(9) $$k^{T}\left(x-a_{i}\right)\approx ∥x-a∥\cos\left(\alpha_{i}\right),\;\;\mathrm{for}\;\;i=1,\ldots ,N,$$

where $\lambda_{i}=10^{\frac{P_{i}}{10\gamma }}$, $\eta =10^{\frac{P_{0}}{10\gamma }}$, $c_{i}={\left[-\sin\left(\phi_{i}\right),\cos\left(\phi_{i}\right),0\right]}^{T}$ and $k={\left[0,0,1\right]}^{T}$.

By following the LS principle and according to (7), (8) and 9, the target location estimate was obtained by solving the following problem:

(10) $$\begin{array}{c} \hat{x}=\underset{x}{\arg\;\min}\sum_{i=1}^{N}{\left(\lambda_{i}∥x-a_{i}∥-\eta d_{0}\right)}^{2}+\sum_{i=1}^{N}{\left(c_{i}^{T}\left(x-a_{i}\right)\right)}^{2}\\ +\sum_{i=1}^{N}{\left(k_{i}^{T}\left(x-a_{i}\right)-∥x-a_{i}∥\;\cos\left(\alpha_{i}\right)\right)}^{2}. \end{array}$$

Obviously, the problem in (10) is still non-convex, and its solution cannot be given in closed-form. To convexify (10), first, the authors in [14] introduced auxiliary variables $r_{i}=∥x-a_{i}∥$, $z=[z_{i}]$ ($z\in R^{3N}$), where $z_{i}=\lambda_{i}∥x-a_{i}∥-\eta d_{0}$, for i=1,…,N, $z_{i}=c_{i}^{T}\left(x-a_{i}\right)$, for i=N+1,…,2N, and $z_{i}=k_{i}^{T}\left(x-a_{i}\right)-∥x-a_{i}∥\;\cos\left(\alpha_{i}\right)$, for i=2N+1,…,3N. This yielded:

(11) $$\begin{array}{c} \underset{x,r,z}{\mathrm{minimize}}\;\;{∥z∥}^{2}\\ \;\mathrm{subject}\;\mathrm{to}:\\ r_{i}=∥x-a_{i}∥,\;\;i=1,\ldots ,N\\ z_{i}=\lambda_{i}r_{i}-\eta d_{0},\;\;i=1,\ldots ,N,\\ z_{i}=c_{i}^{T}\left(x-a_{i}\right),\;\;i=N+1,\ldots ,2N,\\ z_{i}=k_{i}^{T}\left(x-a_{i}\right)-r_{i}\;\cos\left(\alpha_{i}\right),\;\;i=2N+1,\ldots ,3N. \end{array}$$

By introducing an epigraph variable and applying semidefinite cone constraint relaxation, the following problem was obtained.

(12) $$\begin{array}{c} \underset{x,r,z,t}{\mathrm{minimize}}\;\;t\\ \;\mathrm{subject}\;\mathrm{to}:\\ ∥x-a_{i}∥\leq r_{i},\;\;i=1,\ldots ,N\\ z_{i}=\lambda_{i}r_{i}-\eta d_{0},\;\;i=1,\ldots ,N,\\ z_{i}=c_{i}^{T}\left(x-a_{i}\right),\;\;i=N+1,\ldots ,2N,\\ z_{i}=k_{i}^{T}\left(x-a_{i}\right)-r_{i}\;\cos\left(\alpha_{i}\right),\;\;i=2N+1,\ldots ,3N,\\ ∥\left[\begin{array}{cc} I_{3N} & z\\ z & t \end{array}\right]∥⪰0_{3N+1}. \end{array}$$

The problem in (12) is an SDP problem. This type of problem can be solved efficiently by the CVX package [65]. Notice that the constraint ${∥z∥}^{2}\leq t$ was rewritten as a semidefinite cone constraint form by applying the Schur complement [66]. In the further text, the estimator in (12) is referred to as “SDP”.

### 2.3. SOCP Estimator

Note that writing the constraint ${∥z∥}^{2}\leq t$ into a semidefinite cone constraint increases the computational complexity of an algorithm, i.e., its execution time. In [52], it was shown that this constraint, as well as the LS estimator in (10) can be rewritten as an SOCP, which significantly reduces the computational complexity. To this end, first, the authors in [52] introduced auxiliary variables $r_{i}=∥x-a_{i}∥$, $z=[z_{i}]$, $g=[g_{i}]$ and $h=[h_{i}]$, where $z_{i}=\lambda_{i}∥x-a_{i}∥-\eta d_{0}$, $g_{i}=c_{i}^{T}\left(x-a_{i}\right)$, and $h_{i}=k_{i}^{T}\left(x-a_{i}\right)-∥x-a_{i}∥\;\cos\left(\alpha_{i}\right)$, for i=1,…,N. This yielded:

(13) $$\begin{array}{c} \underset{x,r,z,g,h}{\mathrm{minimize}}{\;\;∥z∥}^{2}+{∥g∥}^{2}+{∥h∥}^{2}\\ \;\mathrm{subject}\;\mathrm{to}:\\ r_{i}=∥x-a_{i}∥,\;\;i=1,\ldots ,N\\ z_{i}=\lambda_{i}r_{i}-\eta d_{0},\;\;i=1,\ldots ,N,\\ g_{i}=c_{i}^{T}\left(x-a_{i}\right),\;\;i=1,\ldots ,N,\\ h_{i}=k_{i}^{T}\left(x-a_{i}\right)-r_{i}\;\cos\left(\alpha_{i}\right),\;\;i=1,\ldots ,N. \end{array}$$

Then, epigraph variables $t_{1}$, $t_{2}$ and $t_{3}$ were introduced. By applying second-order cone constraint relaxation, the following problem was obtained.

(14) $$\begin{array}{c} \underset{x,r,z,g,h,t_{1},t_{2},t_{3}}{\mathrm{minimize}}t_{1}+t_{2}+t_{3}\\ \;\mathrm{subject}\;\mathrm{to}\\ ∥x-a_{i}∥\leq r_{i},\;\;i=1,\ldots ,N\\ z_{i}=\lambda_{i}r_{i}-\eta d_{0},\;\;i=1,\ldots ,N,\\ g_{i}=c_{i}^{T}\left(x-a_{i}\right),\;\;i=1,\ldots ,N,\\ h_{i}=k_{i}^{T}\left(x-a_{i}\right)-r_{i}\;\cos\left(\alpha_{i}\right),\;\;i=1,\ldots ,N,\\ ∥\left[\begin{array}{c} 2z\\ t_{1}-1 \end{array}\right]∥\leq t_{1}+1,∥\left[\begin{array}{c} 2g\\ t_{2}-1 \end{array}\right]∥\leq t_{2}+1,∥\left[\begin{array}{c} 2h\\ t_{3}-1 \end{array}\right]∥\leq t_{3}+1. \end{array}$$

The problem in (14) is an SOCP problem, which is readily solved by the CVX package [65]. The main difference between this estimator and the one given in (12) is that the SOCP relaxation technique was applied in (14), whereas the SDP relaxation technique was used in (12) to convexify the derived non-convex estimators. In the further text, (14) is referred to as “SOCP”.

### 2.4. SR-WLS Estimator

Although the computational complexity is significantly decreased by the derived SOCP estimator in (14), it is still relatively high and can be further reduced. Hence, a linear estimator for solving the ML problem was proposed in [54], solved by means of a bisection procedure.

Notice that (7) could also be written as:

(15) $$\zeta_{i}^{2}{∥x-a_{i}∥}^{2}\approx d_{0}^{2},$$

where $\zeta_{i}=10^{\frac{P_{i}-P_{0}}{10\gamma }}$.

**Figure 8:**
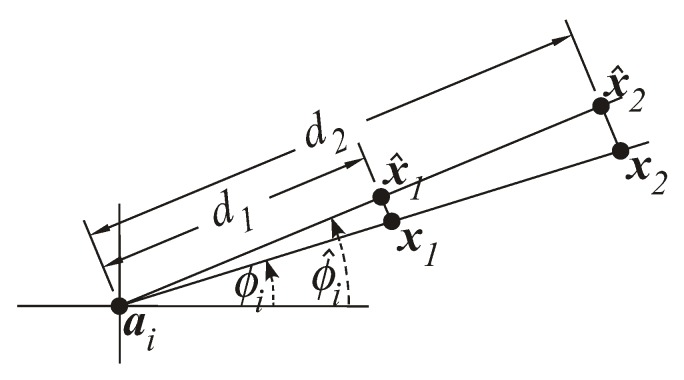
Illustration of azimuth angle measurement error for different distances.

Both RSS and AoA short-distance links are trusted more than the remote ones, due to their multiplicative and additive factors [26]. Thus, in order to enhance the localization accuracy, in (15), the authors in [54] introduced weights, $w=[\sqrt{w_{i}}]$, where each $w_{i}$ was defined as:

(16) $$w_{i}=1-\frac{{\hat{d}}_{i}}{\sum_{i=1}^{N}{\hat{d}}_{i}},$$

with ${\hat{d}}_{i}=d_{0}10^{\frac{P_{0}-P_{i}}{10\gamma }}$ being the ML estimate of the distance obtained from (2). To be more specific, the RSS observations have a constant multiplicative factor with range [26]. This results in a larger error for remote connections in comparison with the nearby ones. In order to give a justification for the weight introduction for AoA observations, the reader is referred to Figure 8.

Figure 8 illustrates the true and the measured azimuth angles, $\phi_{i}$ and ${\hat{\phi }}_{i}$, between an anchor and two targets, $x_{1}$ and $x_{2}$, located along the same direction, but unequally distant from the anchor. The objective is to estimate the locations of the two targets based on the available information. Obviously, the location estimates of the two targets are at points ${\hat{x}}_{1}$ and ${\hat{x}}_{2}$. Nevertheless, Figure 8 shows that the estimated location of the target physically closer to the anchor, ${\hat{x}}_{1}$, is much more accurate than the one further away. In other words, for a given angle, the more two sensors are physically further apart from each other, the greater the set of all possible solutions will be (more likely to impair the localization accuracy).

Then, by replacing $∥x-a_{i}∥$ with ${\hat{d}}_{i}$ in (9) and using weights, a WLS problem was derived according to (15), (8) and modified (9) as:

(17) $$\begin{array}{c} \hat{x}=\underset{x}{\arg\;\min}\sum_{i=1}^{N}w_{i}{\left(\zeta_{i}^{2}{∥x-a_{i}∥}^{2}-d_{0}^{2}\right)}^{2}+\sum_{i=1}^{N}w_{i}{\left(c_{i}^{T}\left(x-a_{i}\right)\right)}^{2}\\ +\sum_{i=1}^{N}w_{i}{\left(k_{i}^{T}\left(x-a_{i}\right)-{\hat{d}}_{i}\;\cos\left(\alpha_{i}\right)\right)}^{2}. \end{array}$$

Similarly to (10), the LS estimator in (17) is not convex. Nonetheless, it was shown in [54] that (17) can be expressed as a quadratic programming problem whose exact solution can be obtained readily [67]. By substituting $y=[x^{T},{∥x∥}^{2}{]}^{T}$, the problem in (17) was reformulated as:

$$\underset{y}{\mathrm{minimize}}\;\;{∥W\left(Ay-b\right)∥}^{2}$$

                    subject to

(18) $$y^{T}Dy+2l^{T}y=0,$$

where $W=I_{3}⊗\mathrm{diag}\left(w\right)$, with ⊗ denoting the Kronecker product,

$$A=\left[\begin{array}{cc} -2\zeta_{1}^{2}a_{1}^{T} & \zeta_{1}^{2}\\ \vdots & \vdots \\ -2\zeta_{N}^{2}a_{N}^{T} & \zeta_{N}^{2}\\ c_{1}^{T} & 0\\ \vdots & \vdots \\ c_{N}^{T} & 0\\ k_{1}^{T} & 0\\ \vdots & \vdots \\ k_{N}^{T} & 0 \end{array}\right],b=\left[\begin{array}{c} d_{0}^{2}-\zeta_{1}^{2}{∥a_{1}∥}^{2}\\ \vdots \\ d_{0}^{2}-\zeta_{N}^{2}{∥a_{N}∥}^{2}\\ c_{1}^{T}a_{1}\\ \vdots \\ c_{N}^{T}a_{N}\\ k_{1}^{T}a_{1}+{\hat{d}}_{1}\cos\left(\alpha_{1}\right)\\ \vdots \\ k_{N}^{T}a_{N}+{\hat{d}}_{N}\cos\left(\alpha_{N}\right) \end{array}\right],D=\left[\begin{array}{cc} I_{q} & 0_{q\times 1}\\ 0_{1\times q} & 0 \end{array}\right],\;\;l=\left[\begin{array}{c} 0_{q\times 1}\\ -1/2 \end{array}\right],$$

and $I_{M}$ and $0_{M\times M}$ denote an identity and a zero matrix of size *M*, respectively.

The estimator in (18) is a GTRS [67,68,69,70,71] (requires minimizing a quadratic function over a quadratic constraint). Although non-convex in general, GTRS is strictly decreasing over an easily computed interval [67,68,69,70,71]; hence, obtaining its exact solution is straightforward by merely a bisection procedure. The estimator in (18) is referred to as “SR-WLS” in the remaining text.

### 2.5. WLS Estimator

The estimator given by (18) has linear computational complexity in the number of anchors, but it is executed iteratively, which raises its execution time. To further reduce the computational complexity, the authors in [72] presented a different WLS estimator that does not require iterative execution and whose solution was written in closed-form.

By resorting to spherical coordinates, vector $x-a_{i}$ was expressed as $x-a_{i}=r_{i}u_{i}:r_{i}\geq 0,∥u_{i}∥=1$, fori=1,…,N. The unit vector, $u_{i}$, was defined by exploiting the available AoA observations as $u_{i}={\left[\cos\left(\phi_{i}\right)\sin\left(\alpha_{i}\right),\sin\left(\phi_{i}\right)\sin\left(\alpha_{i}\right),\cos\left(\alpha_{i}\right)\right]}^{T}$. The authors in [72] then applied the described conversion in (7) and (9) and multiplied with $u_{i}^{T}u_{i}$, obtaining respectively:

(19) $$\lambda_{i}u_{i}^{T}r_{i}u_{i}\approx \eta d_{0}\Leftrightarrow \lambda_{i}u_{i}^{T}\left(x-a_{i}\right)\approx \eta d_{0}$$

(20) $$k_{i}^{T}r_{i}u_{i}\approx u_{i}^{T}r_{i}u_{i}\cos\left(\alpha_{i}\right)\Leftrightarrow {\left(\cos\left(\alpha_{i}\right)u_{i}-k_{i}\right)}^{T}\left(x-a_{i}\right)\approx 0$$

where the symbol ⇔ is used to denote equivalence.

According to the WLS criterion and by taking advantage of (8), (16), (19) and (20), the following estimator was obtained.

(21) $$\begin{array}{c} \hat{x}=\underset{x}{\arg\;\min}\sum_{i=1}^{N}w_{i}{\left(\lambda_{i}u_{i}^{T}\left(x-a_{i}\right)-\eta d_{0}\right)}^{2}\\ +\sum_{i=1}^{N}w_{i}{\left(c_{i}^{T}\left(x-a_{i}\right)\right)}^{2}+\sum_{i=1}^{N}w_{i}{\left({\left(\cos\left(\alpha_{i}\right)u_{i}-k_{i}\right)}^{T}\left(x-a_{i}\right)\right)}^{2}. \end{array}$$

The WLS in (21) was then rewritten in an equivalent vector form, i.e.,

(22) $$\underset{x}{\mathrm{minimize}}\;\;{∥W\left(\tilde{A}x-\tilde{b}\right)∥}^{2}$$

where:

$$\tilde{A}=\left[\begin{array}{c} \rho_{1}u_{1}^{T}\\ \vdots \\ \rho_{1}u_{N}^{T}\\ c_{1}^{T}\\ \vdots \\ c_{N}^{T}\\ {\left(\cos\left(\alpha_{1}\right)u_{1}-k_{1}\right)}^{T}\\ \vdots \\ {\left(\cos\left(\alpha_{N}\right)u_{N}-k_{N}\right)}^{T} \end{array}\right],\;\tilde{b}=\left[\begin{array}{c} \rho_{1}u_{1}^{T}a_{1}+\eta d_{0}\\ \vdots \\ \rho_{N}u_{N}^{T}a_{N}+\eta d_{0}\\ c_{1}^{T}a_{1}\\ \vdots \\ c_{N}^{T}a_{N}\\ {\left(\cos\left(\alpha_{1}\right)u_{1}-k_{1}\right)}^{T}a_{1}\\ \vdots \\ {\left(\cos\left(\alpha_{N}\right)u_{N}-k_{N}\right)}^{T}a_{N} \end{array}\right].$$

The closed-form solution to (22) is readily given by:

$$\hat{x}={\left({\tilde{A}}^{T}W^{T}W\tilde{A}\right)}^{-1}\left({\tilde{A}}^{T}W^{T}\tilde{b}\right).$$

In the remaining text, we refer to the estimator in (22) as “WLS”.

### 2.6. LS Estimator

When both distance and angle measurements are available, the target location can be estimated simply by taking a step of size ${\hat{d}}_{i}$ from the *i*-th anchor in a direction given by $\phi_{i}$ and an inclination given by $\alpha_{i}$ [46], i.e.,

(23) $$\begin{array}{c} {\hat{x}}_{x}=a_{ix}+{\hat{d}}_{i}\cos\left(\phi_{i}\right)\sin\left(\alpha_{i}\right),\\ {\hat{x}}_{y}=a_{iy}+{\hat{d}}_{i}\sin\left(\phi_{i}\right)\sin\left(\alpha_{i}\right),\\ {\hat{x}}_{z}=a_{iz}+{\hat{d}}_{i}\cos\left(\alpha_{i}\right),\;\;\;\mathrm{for}\;\;\;i=1,\ldots ,N. \end{array}$$

By reformulating (23) into a vector form and applying the LS estimation, the authors in [46] determined the target location estimate according to:

(24) $$\hat{x}={\left(S^{T}WS\right)}^{-1}S^{T}Wu,$$

where:

$$S=\left[\begin{array}{ccc} e_{N} & 0_{N} & 0_{N}\\ 0_{N} & e_{N} & 0_{N}\\ 0_{N} & 0_{N} & e_{N} \end{array}\right],\;u=\left[\begin{array}{c} a_{1x}+{\hat{d}}_{1}\cos\left(\phi_{1}\right)\sin\left(\alpha_{1}\right)\\ \vdots \\ a_{Nx}+{\hat{d}}_{N}\cos\left(\phi_{N}\right)\sin\left(\alpha_{N}\right)\\ a_{1y}+{\hat{d}}_{1}\sin\left(\phi_{1}\right)\sin\left(\alpha_{1}\right)\\ \vdots \\ a_{Ny}+{\hat{d}}_{N}\sin\left(\phi_{N}\right)\sin\left(\alpha_{N}\right)\\ a_{1z}+{\hat{d}}_{1}\cos\left(\alpha_{1}\right)\\ \vdots \\ a_{Nz}+{\hat{d}}_{N}\cos\left(\alpha_{N}\right) \end{array}\right].$$

with $e_{N}$ and $0_{N}$ denoting column vectors of *N* ones and zeros, respectively. This estimator offers a relatively accurate solution, but its performance can still be enhanced by introducing weights.

The estimator in (24) is referred to as “LS” in the further text.

### 2.7. WLLS Estimator

In [73], the authors used a similar approach with the one presented in [46]. The main difference however was that the authors in [73] incorporated weights into their algorithm and considered the path loss exponent as an unknown variable, as well. They started by rewriting the measurement model (2) into a slightly different form, i.e.,

(25) $$P_{i}=P_{0}-\nu \gamma \ln∥x-a_{i}∥+n_{i},$$

where $\nu =\frac{10}{\ln(10)}$ and the fact that $d_{0}=1$ m was used, as well. Then, the distance estimates in [73] were obtained in the following bias-corrected manner.

(26) $${\tilde{d}}_{i}={\hat{d}}_{i}\exp\left\{-\frac{\sigma_{n_{i}}^{2}}{2{\left(\gamma \nu \right)}^{2}}\right\}\kappa_{i},\;\;\;\mathrm{for}\;\;\;i=1,\ldots ,N,$$

where $\kappa_{i}=\exp\left\{-\frac{\sigma_{n_{i}}^{2}}{2{\left(\nu \gamma \right)}^{2}}\right\}$ was defined as an unbiasing constant for RSS measurement [1].

Then, the solution to the localization problem was given as:

(27) $$\hat{x}={\left(S^{T}C^{-1}S\right)}^{-1}S^{T}C^{-1}\tilde{u},$$

where:

$$\tilde{u}=\left[\begin{array}{c} a_{1x}+{\tilde{d}}_{1}\cos\left(\phi_{1}\right)\sin\left(\alpha_{1}\right)\delta_{1}\\ \vdots \\ a_{Nx}+{\tilde{d}}_{N}\cos\left(\phi_{N}\right)\sin\left(\alpha_{N}\right)\delta_{N}\\ a_{1y}+{\tilde{d}}_{1}\sin\left(\phi_{1}\right)\sin\left(\alpha_{1}\right)\delta_{1}\\ \vdots \\ a_{Ny}+{\tilde{d}}_{N}\sin\left(\phi_{N}\right)\sin\left(\alpha_{N}\right)\delta_{N}\\ a_{1z}+{\tilde{d}}_{1}\cos\left(\alpha_{1}\right){\tilde{\delta }}_{1}\\ \vdots \\ a_{Nz}+{\tilde{d}}_{N}\cos\left(\alpha_{N}\right){\tilde{\delta }}_{N} \end{array}\right],\;C=\left[\begin{array}{ccc} C_{\mathrm{xx}} & C_{\mathrm{xy}} & C_{\mathrm{xz}}\\ C_{\mathrm{yx}} & C_{\mathrm{yy}} & C_{\mathrm{yz}}\\ C_{\mathrm{zx}} & C_{\mathrm{zy}} & C_{\mathrm{zz}} \end{array}\right],$$

with $\delta_{i}=\exp\left\{\frac{\sigma_{v_{i}}^{2}+\sigma_{m_{i}}^{2}}{2}-\frac{\sigma_{n_{i}}^{2}}{2{\left(\gamma \nu \right)}^{2}}\right\}$, ${\tilde{\delta }}_{i}=\exp\left\{\frac{\sigma_{v_{i}}^{2}}{2}-\frac{\sigma_{n_{i}}^{2}}{2{\left(\gamma \nu \right)}^{2}}\right\}$, and the elements of the covariance matrix C are given in Appendix A. Although this approach offers very good estimation accuracy, it depends on perfect knowledge of the noise standard deviations ($\sigma_{n_{i}},\sigma_{m_{i}}$ and $\sigma_{v_{i}}$), which might not be feasible in practice.

The estimator in (27) is referred to as “WLLS” in the remaining text.

## 3. Complexity Analysis

The trade-off between the localization accuracy and the computational complexity is one of the key attributes of any algorithm because it directly determines its applicability potential. Therefore, apart from the performance, one needs to compare the computational complexities of any two estimators, as well. Therefore, this section summarizes the computational complexities of the approaches described in Section 2.

The worst case computational complexity of a mixed SDP/SOCP is given by [74]:

(28) $$O\left(\sqrt{L}\left(m\sum_{i=1}^{N_{sd}}{\left(n_{i}^{sd}\right)}^{3}+m^{2}\sum_{i=1}^{N_{sd}}{\left(n_{i}^{sd}\right)}^{2}+m^{2}\sum_{i=1}^{N_{soc}}n_{i}^{soc}+\sum_{i=1}^{N_{soc}}{\left(n_{i}^{soc}\right)}^{2}+m^{3}\right)\right)\;,$$

where *L* is the iteration complexity of the algorithm, *m* is the number of equality constraints, $n_{i}^{sd}$ and $n_{i}^{soc}$ are respectively the dimensions of the *i*-th semidefinite cone (SDC) and the *i*-th second-order cone (SOC) and $N_{i}^{sd}$ and $N_{i}^{soc}$ are the number of SDC and SOC constraints, respectively. The formula in (28) corresponds to the formula for computing the complexity of an SDP for the case when we have no SOCCs (in which case, *L* is the dimension of the SDC given as a result of accumulating all SDC), and vice versa (in which case *L* is the total number of SOC constraints) [74]. Hence, it is used to analyze the complexities of the considered algorithms in this paper.

Assuming that *K* is the maximum number of steps in the bisection procedure used to solve (18), Table 1 provides an overview of the considered algorithms together with their worst case computational complexities.

Table 1 shows that the computational burden of all methods depends principally on the network size, i.e., the number of anchors in the network. This feature is common to methods operating in a centralized manner [54], since all information is transferred to a central node (processor). Table 1 shows that the optimization-based algorithms have the highest computational complexity, while the remaining algorithms have linear computational cost in *N*. However, owing to its iterative nature, the SR-WLS algorithm has slightly higher computational burden in comparison with the rest of the linear algorithms.

## 4. Performance Results

This section presents a set of numerical results in order to compare the performance of the algorithms described in Section 2 via computer simulations. All radio observations were generated by using (2)–(4). A random deployment of all inside a box of edge length B=15 m in each Monte Carlo, $M_{c}$, run was considered. As it was considered in most existing works, here, the reference distance is set to $d_{0}=1$ m, the reference path loss to $P_{0}=-10$ dBm and the PLE fixed as γ=2.5. Nonetheless, perfect knowledge of the PLE is virtually impossible to obtain in practice. Hence, in order to introduce a somewhat more realistic measurement model mismatch, and at the same time test the robustness of the considered algorithms to imperfect knowledge of the PLE, the true value of the PLE was drawn from a uniform distribution on an interval [2.2,2.8], i.e., $\gamma_{i}\in U\left[2.2,2.8\right]$ for i=1,…,N. Moreover, K=30 is used for the SR-WLS method in (18). The main performance metric used here is the root mean square error (RMSE), defined as $\mathrm{RMSE}=\sqrt{\sum_{i=1}^{M_{c}}\frac{∥x_{i}-{\hat{x}}_{i}{∥}^{2}}{M_{c}}},$ where ${\hat{x}}_{i}$ is used to denote the estimate of the true target location, $x_{i}$, in the *i*-th $M_{c}$ run. Finally, it is worth mentioning that the results of the Cramer–Rao lower bound (CRLB) for hybrid RSS/AoA localization, derived in Appendix B, are also included in all figures here. Nonetheless, since the RSS-based estimators are biased in general [26], this bound is just a theoretical one and is shown here for the sake of completeness only.

**Figure 9:**
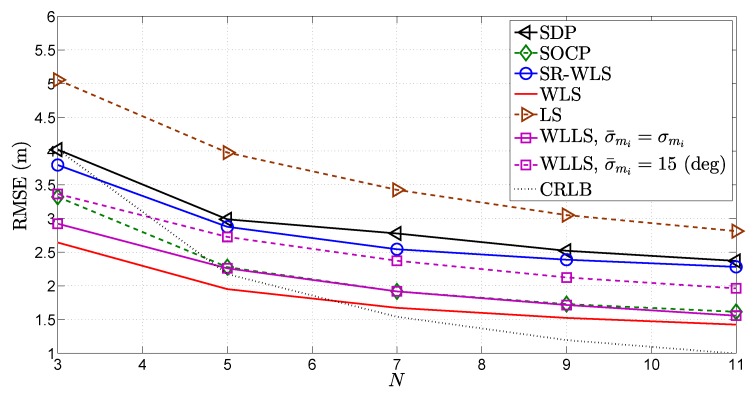
RMSE (m) versus *N* comparison, when $\sigma_{n_{i}}=6$ dB, $\sigma_{m_{i}}=10$ deg, $\sigma_{v_{i}}=10$ deg, $\gamma_{i}\in U\left[2.2,2.8\right]$, γ=2.5, B=15 m, $P_{0}=-10$ dBm, $d_{0}=1$ m, $M_{c}=50,000$.

Figure 9 illustrates the RMSE (m) versus *N* comparison. In order to show the influence of imperfect knowledge about the noise powers on the performance of the WLLS method, the results for WLLS when the assumed noise power of the azimuth angle, ${\bar{\sigma }}_{m_{i}}$, is fixed to a value different from the true one are also presented. From Figure 9, it can be seen that all algorithms profit from the extra information added by incrementing *N*, as expected. Nevertheless, the WLS algorithm shows superior performance for all *N*. This is an important result, since the computational complexity of WLS is linear with *N*, unlike the complexity of SOCP and SDP. It is also important to highlight the fact that the noise powers in Figure 9 were set to a relatively high value and that even in such a setting, all methods behave relatively well.

**Figure 10:**
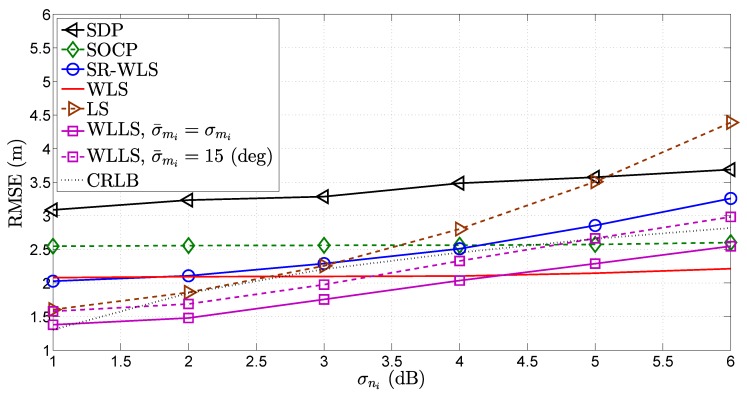
RMSE (m) versus $\sigma_{n_{i}}$ (dB) comparison, when N=4, $\sigma_{m_{i}}=10$ deg, $\sigma_{v_{i}}=10$ deg, $\gamma_{i}\in U\left[2.2,2.8\right]$, γ=2.5, B=15 m, $P_{0}=-10$ dB, $d_{0}=1$ m, $M_{c}=50,000$.

**Figure 11:**
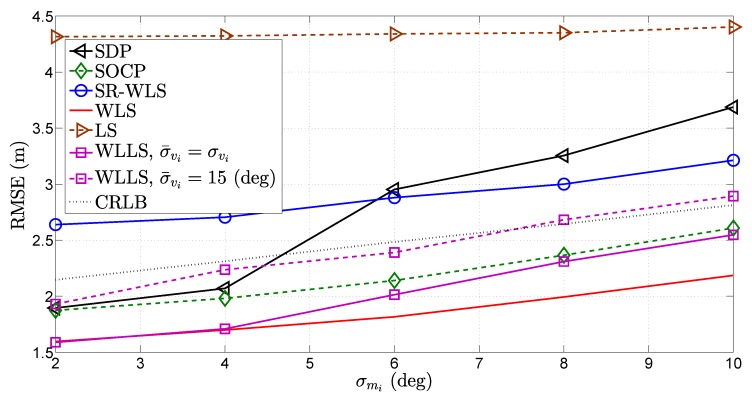
RMSE (m) versus $\sigma_{m_{i}}$ (deg) comparison, when N=4, $\sigma_{n_{i}}=6$ dB, $\sigma_{v_{i}}=10$ deg, $\gamma_{i}\in U\left[2.2,2.8\right]$, γ=2.5, B=15 m, $P_{0}=-10$ dBm, $d_{0}=1$ m, $M_{c}=50,000$.

**Figure 12:**
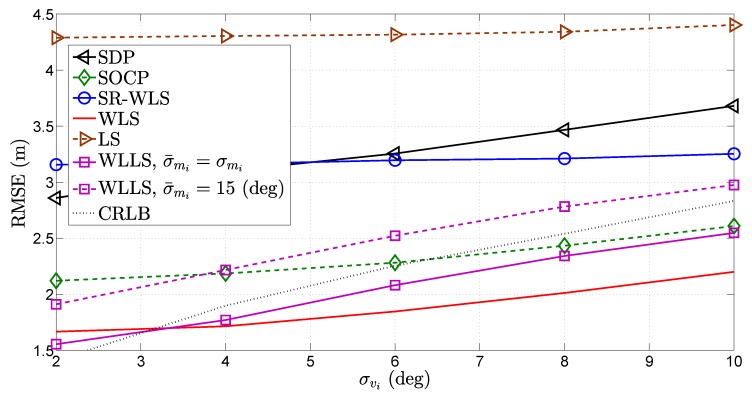
RMSE (m) versus $\sigma_{v_{i}}$ (deg) comparison, when N=4, $\sigma_{n_{i}}=6$ dB, $\sigma_{m_{i}}=10$ deg, $\gamma_{i}\in U\left[2.2,2.8\right]$, γ=2.5, B=15 m, $P_{0}=-10$ dBm, $d_{0}=1$ m, $M_{c}=50,000$.

In Figure 10, Figure 11 and Figure 12, the influence of the quality of different types of measurements on the performance of the considered approaches was investigated. More precisely, Figure 10, Figure 11 and Figure 12 illustrate the RMSE (m) versus $\sigma_{n_{i}}$ (dB), $\sigma_{m_{i}}$ (deg) and $\sigma_{v_{i}}$ (deg) comparison, respectively, for N=4. As anticipated, Figure 10, Figure 11 and Figure 12 show that the performance of all algorithms deteriorates as the quality of any measurement decreases. Nevertheless, it can be noticed that different measurements have a different influence on the performance of the considered algorithms. For instance, the WLS algorithm suffers small impairments as the quality of the RSS measurement deteriorates, while the the same cannot be said about the influence of the quality of the AoA measurements. This fact is not entirely surprising, since this algorithm is derived based on Cartesian to spherical coordinate conversion, where the AoA measurements play the key roll. However, it can be said that the deteriorations suffered by any algorithm are mild for such a wide span of noise power, and all algorithms perform relatively well.

**Figure 13:**
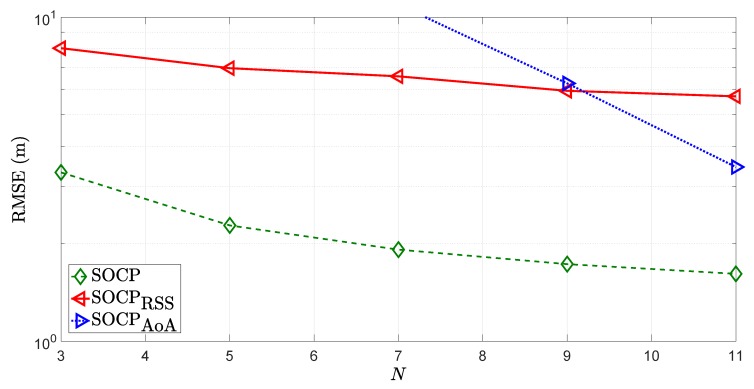
RMSE (m) versus *N* comparison, when $\sigma_{n_{i}}=6$ dB, $\sigma_{m_{i}}=10$ deg, $\sigma_{v_{i}}=10$ deg, $\gamma_{i}\in U\left[2.2,2.8\right]$, γ=2.5, B=15 m, $P_{0}=-10$ dBm, $d_{0}=1$ m, $M_{c}=10,000$.

Lastly, a comparison between a hybrid system versus the classical ones, based on RSS-only and AoA-only measurements is presented in Figure 13. The figure illustrates the RMSE (m) versus *N* comparison of the SOCP method described in Section 2.3 against its counterparts using RSS-only and AoA-only measurements, denoted by ${\mathrm{SOCP}}_{\mathrm{RSS}}$ and ${\mathrm{SOCP}}_{\mathrm{AoA}}$, respectively. From Figure 13, it is clear that an estimator can benefit significantly from the measurement integration, especially in the case of low *N*. Furthermore, hybrid estimators can even provide a solution with only one measurement in three-dimensional scenarios, while the classical ones require at least two measurements (RSS-only estimators require at least three measurements).

In this section, the performance comparison of the considered algorithms was done based on simulation results, exclusively. For a comparison based on real indoor experimental data, the reader is referred to [75,76,77]. In these works, the authors used WiFi technology to acquire the RSS and AoA measurements. More precisely, a regular 802.11-equipped laptop took four sets of measurements at each measurement point, one for each pose of the target (facing north, east, south and west). The way that AoA measurements were extracted from an 802.11 base station was that a directional antenna was attach to a wireless access point. When this antenna was rotated, the RSS reported by the card was higher in the direction of the measurement point in general (in roughly 90% of the cases, it came from the first two peaks). To automatize this measurement of the angle, the authors mounted a small Toshiba Libretto 70ct laptop on a record player (turntable). In order to obtain a higher difference in the maximums, an antenna that is highly directional was chosen. The Lucent 2 Mbps 802.11 card was linked to a Hyperlink 14-dB gain directional antenna. The antenna was attached to the bottom of the laptop, so that it rotated in the horizontal plane. Such real indoor measurements were then utilized for comparison purposes in [75,76,77]. The findings presented in [75,76,77] are in concordance with the ones shown here, i.e., the hybrid systems show superior performance in comparison with the classical ones.

## 5. Conclusions and Future Work

This section summarizes the main conclusions and attained results of this work (Section 5.1) and discusses, in further detail, some foreseen directions for future research on the main topic (Section 5.2).

### 5.1. Conclusions

In this work, the problem of target localization in WSN by using hybrid RSS/AoA measurements was addressed. A set of very recently-developed localization algorithms was presented and analyzed in detail. A common objective of the considered localization algorithms is to estimate the unknown location of the target by solving a tight approximation of the original problem, which represents an excellent framework even under inopportune network configuration and strong measurement noise. A strong emphasis was made on convex relaxations and derivation of convex problems, whose global minima can be readily obtained through general-purpose solvers. Moreover, the solution obtained through the algorithms could also be used as an initial point for iterative methods, in which case the risk of convergence to local minima of these methods is minimized and near-optimal performance could be obtained. The presented algorithms are relatively easy to implement, and they offer good estimation accuracy in a single iteration.

A set of simulation results was presented in this work, together with a detailed analysis of the computational complexity. The simulation results corroborate the effectiveness of the presented algorithms, which represent an excellent trade-off between the estimation accuracy and the computational complexity. Various network configurations were studied for a broad spectra of parameter settings, and in all of them, the presented algorithms showed good performance and robustness to not knowing additional parameters (such as the PLE), beyond the target location.

### 5.2. Future Work

Even though much has been done in the area of RSS/AoA target localization, there is still a number of possibilities for future research. One interesting direction for future research might be the development of new algorithms and adaptation of the presented algorithms to a more challenging scenarios of indoor localization in severe NLoS environments. NLoS can significantly degrade the localization accuracy, especially in the case where the configuration of the environment is not known, i.e., when it is not known a priori which links are line-of-sight (LoS) and which are NLoS. Instead of trying to distinguish between LoS and NLoS links and disregarding the NLoS ones, because there is always a probability of false alarm or false detection, it would be of interest to exploit the property of positive NLoS bias, which is known to be much larger than the measurement noise.

In this work, a constant network topology during the computational phase was taken for granted. A more realistic scenario, where sensors and/or links can fail with a certain probability might be of interest in some applications, especially for distributed algorithms, which are carried out in an iterative fashion. Such a problem would represent a serious challenge for any localization algorithm, as it could lead to network disconnection or even isolated islands of sensors with no or very scarce information, insufficient for good location estimation.

Similar to the last possibility, in large-scale WSNs, it might be of interest to investigate the case where targets limit the number of cooperating nodes. In the case where a target has a high number of neighbors, selecting only a certain number of its neighbors might be of interest by, e.g., choosing only the nearest ones such that the computational burden is decreased and that its estimation accuracy is unaffected, or possibly even further improved (in the case where one or more very noise links (outliers) are disregarded). The main challenge in such a problem would be to design an intelligent neighbor-selecting strategy, owing to noisy observations that might mislead a target to disregard a potentially good link and maintain a bad one.

In the case of distributed algorithm execution, the design of simple MAC protocols, such as a second-order coloring scheme [33], could be interesting as it might lead to error and time-execution reduction. By designing a more intelligent routine for the operating hierarchy (e.g., such that targets with the highest number of anchor neighbors work first) might produce better estimation accuracy and at the same time increase the convergence rate of an algorithm, since one would expect to obtain a better estimation for those targets that might propagate inside the network.

Another possible direction for future research might be target tracking and/or navigation. The algorithms presented here only made use of radio measurements and disregarded any prior knowledge that might be obtained in the localization process. Therefore, an interesting research topic for the future might be to consider real-time localization of a moving target by integrating any prior knowledge that might be gathered during this process into an estimator [78,79]. Furthermore, by knowing the terrain configuration and by tracking the location of a mobile target, relatively accurate target navigation schemes could be developed. Such an application might be of practical interest in search and rescue missions, exploration in hostile environments and robotics.

All of the existing works assume omnidirectional antenna directivity such that the set of all possible solutions belongs to the area formed by an intersection of multiple circle-shaped contours. Although the presented methods work well in all considered scenarios, this assumption might be an oversimplification of the problem, since the antenna radiation pattern is non-isotropic in practice (e.g., the antenna radiation pattern depends on the antenna geometry configuration: shape and dimension, dielectric material, combination (antenna array) and signal wavelength); therefore, in practice, the area accommodating the target formed by the intersection of non-circular power contours determined by the antenna pattern. Hence, there seems to be some room for further improvement of the presented algorithms by taking the antenna pattern into consideration when deriving a localization scheme [80].

Furthermore, it might be of great practical interest to adapt the existing or develop novel estimators for a particular application, for instance for AAL [23] or smart indoor positioning systems for situation awareness in emergency situations [24,81]. AAL is a growing research area whose main goal is to create better life and healthcare conditions for older people and/or people with disabilities, by building a flexible set of basic and task-oriented services [23]. Integration of the accurate location of people and/or objects into such services can significantly improve safety and efficiency in everyday life (e.g., assistance for the elderly or people with disabilities, to identify patterns of movements and trigger alerts when anomalies are detected, smart parking, monitoring of storage conditions and goods, navigation-aid for the visually impaired, guidance through shopping malls, airports, etc., workforce management, finding nearby emergency services, etc.). To this end, building a smartphone application, for instance based on accurate indoor localization, might be of great importance and help for both primary users (elders and people with disabilities) and caregivers [82].

Finally, the work studied the target localization problem by using combined RSS and AoA measurements. Employing other types of measurements such as ToA, TDoA, frequency or phase of arrival, to name a few, or a combination of them to solve the localization problem might be of interest for future research, as well.

## Figures and Tables

**Table 1:** Summary of the considered algorithms. SDP, semidefinite programming; SOCP, second-order cone programming; SR-WLS, squared-range weighted LS.

Algorithm	Description	Complexity
SDP	The SDP algorithm in (12)	$O\left(N^{4.5}\right)$
SOCP	The SOCP algorithm in (14)	$O\left(N^{3.5}\right)$
SR-WLS	The SR-WLS algorithm in (18)	$O\left(KN\right)$
WLS	The WLS algorithm in (22)	$O\left(N\right)$
LS	The LS algorithm in (24)	$O\left(N\right)$
WLLS	The WLLS algorithm in (27)	$O\left(N\right)$

## Appendix A. Derivation of the Covariance Matrix

The elements of the covariance matrix C in (27) are given as:

$$\begin{array}{c} C_{\mathrm{xx}}=\mathrm{diag}\left\{\left[\frac{{\tilde{d}}_{i}^{2}}{4}\exp\left\{\frac{\sigma_{n_{i}}^{2}}{{\left(\gamma \nu \right)}^{2}}+\sigma_{m_{i}}^{2}+\sigma_{v_{i}}^{2}\right\}-\frac{{\tilde{d}}_{i}^{2}}{4}\cos\left(2\alpha_{i}\right)\exp\left\{\frac{\sigma_{n_{i}}^{2}}{{\left(\gamma \nu \right)}^{2}}+\sigma_{m_{i}}^{2}-\sigma_{v_{i}}^{2}\right\}\right.\right.\\ \left.\left.+\frac{{\tilde{d}}_{i}^{2}}{4}\cos\left(2\phi_{i}\right)\exp\left\{\frac{\sigma_{n_{i}}^{2}}{{\left(\gamma \nu \right)}^{2}}-\sigma_{m_{i}}^{2}+\sigma_{v_{i}}^{2}\right\}-\frac{{\tilde{d}}_{i}^{2}}{4}\cos\left(2\phi_{i}\right)\cos\left(2\alpha_{i}\right)\exp\left\{\frac{\sigma_{n_{i}}^{2}}{{\left(\gamma \nu \right)}^{2}}-\sigma_{m_{i}}^{2}-\sigma_{v_{i}}^{2}\right\}\right.\right.\\ \left.\left.-\left({\tilde{d}}_{i}\cos\left(\phi_{i}\right)\cos\left(\alpha_{i}\right)\right)\right]\right\}, \end{array}$$

$$\begin{array}{c} C_{\mathrm{yy}}=\mathrm{diag}\left\{\left[\frac{{\tilde{d}}_{i}^{2}}{4}\exp\left\{\frac{\sigma_{n_{i}}^{2}}{{\left(\gamma \nu \right)}^{2}}+\sigma_{m_{i}}^{2}+\sigma_{v_{i}}^{2}\right\}-\frac{{\tilde{d}}_{i}^{2}}{4}\cos\left(2\alpha_{i}\right)\exp\left\{\frac{\sigma_{n_{i}}^{2}}{{\left(\gamma \nu \right)}^{2}}+\sigma_{m_{i}}^{2}-\sigma_{v_{i}}^{2}\right\}\right.\right.\\ \left.\left.-\frac{{\tilde{d}}_{i}^{2}}{4}\cos\left(2\phi_{i}\right)\exp\left\{\frac{\sigma_{n_{i}}^{2}}{{\left(\gamma \nu \right)}^{2}}-\sigma_{m_{i}}^{2}+\sigma_{v_{i}}^{2}\right\}+\frac{{\tilde{d}}_{i}^{2}}{4}\cos\left(2\phi_{i}\right)\cos\left(2\alpha_{i}\right)\exp\left\{\frac{\sigma_{n_{i}}^{2}}{{\left(\gamma \nu \right)}^{2}}-\sigma_{m_{i}}^{2}-\sigma_{v_{i}}^{2}\right\}\right.\right.\\ \left.\left.-\left({\tilde{d}}_{i}\sin\left(\phi_{i}\right)\sin\left(\alpha_{i}\right)\right)\right]\right\}, \end{array}$$

$$\begin{array}{c} C_{\mathrm{zz}}=\mathrm{diag}\left\{\left[\frac{{\tilde{d}}_{i}^{2}}{2}\exp\left\{\frac{\sigma_{n_{i}}^{2}}{{\left(\gamma \nu \right)}^{2}}+\sigma_{v_{i}}^{2}\right\}+\frac{{\tilde{d}}_{i}^{2}}{2}\cos\left(2\alpha_{i}\right)\exp\left\{\frac{\sigma_{n_{i}}^{2}}{{\left(\gamma \nu \right)}^{2}}-\sigma_{v_{i}}^{2}\right\}-\left({\tilde{d}}_{i}\cos\left(\alpha_{i}\right)\right)\right]\right\}, \end{array}$$

$$\begin{array}{c} C_{\mathrm{xy}}=C_{\mathrm{yx}}=\mathrm{diag}\left\{\left[\frac{{\tilde{d}}_{i}^{2}}{2}\sin\left(\phi_{i}\right)\cos\left(\phi_{i}\right)\exp\left\{\frac{\sigma_{n_{i}}^{2}}{{\left(\gamma \nu \right)}^{2}}-\sigma_{m_{i}}^{2}+\sigma_{v_{i}}^{2}\right\}\right.\right.\\ \left.\left.-\frac{{\tilde{d}}_{i}^{2}}{2}\sin\left(\phi_{i}\right)\cos\left(\phi_{i}\right)\cos\left(2\alpha_{i}\right)\exp\left\{\frac{\sigma_{n_{i}}^{2}}{{\left(\gamma \nu \right)}^{2}}-\sigma_{m_{i}}^{2}-\sigma_{v_{i}}^{2}\right\}-{\tilde{d}}_{i}^{2}\sin\left(\phi_{i}\right)\cos\left(\phi_{i}\right){\left(\sin\left(\alpha_{i}\right)\right)}^{2}\right]\right\}, \end{array}$$

$$C_{\mathrm{xz}}=C_{\mathrm{zx}}=\mathrm{diag}\left\{\left[{\tilde{d}}_{i}^{2}\cos\left(\phi_{i}\right)\sin\left(\alpha_{i}\right)\cos\left(\alpha_{i}\right)\exp\left\{\frac{\sigma_{n_{i}}^{2}}{{\left(\gamma \nu \right)}^{2}}-\sigma_{v_{i}}^{2}\right\}-{\tilde{d}}_{i}^{2}\cos\left(\phi_{i}\right)\sin\left(\alpha_{i}\right)\cos\left(\alpha_{i}\right)\right]\right\},$$

and:

$$C_{\mathrm{yz}}=C_{\mathrm{zy}}=\mathrm{diag}\left\{\left[{\tilde{d}}_{i}^{2}\sin\left(\phi_{i}\right)\sin\left(\alpha_{i}\right)\cos\left(\alpha_{i}\right)\exp\left\{\frac{\sigma_{n_{i}}^{2}}{{\left(\gamma \nu \right)}^{2}}-\sigma_{m_{i}}^{2}\right\}-{\tilde{d}}_{i}^{2}\sin\left(\phi_{i}\right)\sin\left(\alpha_{i}\right)\cos\left(\alpha_{i}\right)\right]\right\}.$$

## Appendix B. CRLB Derivation for RSS-AoA Localization

CRLB offers a lower bound on the variance of any unbiased estimator. This means that it is physically not possible to derive an unbiased estimator whose variance is less than the bound. Therefore, CRLB provides a benchmark against which the performance of any unbiased estimator can be compared. In the case that an estimator attains the bound for all values of the unknown parameters, such an estimator is called the minimum variance unbiased estimator [64].

Denote by $y={\left[x_{k}^{T},P_{0}\right]}^{T},\;\;\;k=1,\ldots ,M$, the 3M+1 vector of all unknown parameters. The variance of any unbiased estimator is lower bounded by $\mathrm{var}\left(\hat{y}\right)\geq {\left[J^{-1}\left(y\right)\right]}_{ii}$ [64], where J(y) is the (3M+1)×(3M+1) Fisher information matrix (FIM). The elements of the FIM are defined as ${\left[J(y)\right]}_{i,j}=-E\left[\frac{\partial^{2}\ln p\left(\theta |y\right)}{\partial y_{i}\partial y_{j}}\right]$, where i,j=1,…,(3M+1), and p(θ|y) is the joint conditional probability density function of the observation vector $\theta =[P^{T},\phi^{T},\alpha^{T}]$ ($P={\left[P_{ij}^{A},P_{ik}^{B}\right]}^{T}$, $\phi ={\left[\phi_{ij}^{A},\phi_{ik}^{B}\right]}^{T}$, $\alpha ={\left[\alpha_{ij}^{A},\alpha_{ik}^{B}\right]}^{T}$), given y.

Then, the FIM is computed as:

$$J\left(y\right)=\frac{1}{\sigma_{n_{ij}}^{2}}\;\sum_{(i,j):(i,j)\in A}\;h_{ij}h_{ij}^{T}+\frac{1}{\sigma_{m_{ij}}^{2}}\;\sum_{(i,j):(i,j)\in A}\;q_{ij}q_{ij}^{T}+\frac{1}{\sigma_{v_{ij}}^{2}}\;\sum_{(i,j):(i,j)\in A}\;u_{ij}u_{ij}^{T}$$

$$+\frac{1}{\sigma_{n_{ik}}^{2}}\;\sum_{(i,k):(i,k)\in B}\;h_{ik}h_{ik}^{T}+\frac{1}{\sigma_{m_{ik}}^{2}}\;\sum_{(i,k):(i,k)\in B}\;q_{ik}q_{ik}^{T}+\frac{1}{\sigma_{v_{ik}}^{2}}\;\sum_{(i,k):(i,k)\in B}\;u_{ik}u_{ik}^{T},$$

where:

$$h_{ij}=\rho -\frac{10\gamma d_{0}}{\ln(10)}\frac{E_{i}\left(E_{i}^{T}y-a_{j}\right)}{∥E_{i}^{T}y-a_{j}{∥}^{2}},$$

$$q_{ij}=\frac{E_{i}e_{2}\left(e_{1}^{T}E_{i}^{T}y-e_{1}^{T}a_{j}\right)-E_{i}e_{1}\left(e_{2}^{T}E_{i}^{T}y-e_{2}^{T}a_{j}\right)}{{\left(e_{1}^{T}E_{i}^{T}y-e_{1}^{T}a_{j}\right)}^{2}+{\left(e_{2}^{T}E_{i}^{T}y-e_{2}^{T}a_{j}\right)}^{2}},$$

$$u_{ij}=\frac{E_{i}(e_{3}∥E_{i}^{T}y-a_{j}∥-\left(E_{i}^{T}y-a_{j}\right)\left(e_{3}^{T}E_{i}^{T}y-e_{3}^{T}a_{j}\right))}{∥E_{i}^{T}y-a_{j}{∥}^{2}\sqrt{∥E_{i}^{T}y-a_{j}{∥}^{2}-{\left(e_{3}^{T}E_{i}^{T}y-e_{3}^{T}a_{j}\right)}^{2}}},$$

$$h_{ik}=\rho -\frac{10\gamma d_{0}}{\ln(10)}\;\;\frac{\left(E_{i}-E_{k}\right)\left(E_{i}^{T}y-E_{k}^{T}y\right)}{∥E_{i}^{T}y-E_{k}^{T}{y∥}^{2}},$$

$$q_{ik}=\frac{\left(E_{i}-E_{k}\right)\left(e_{2}\left(e_{1}^{T}E_{i}^{T}y-e_{1}^{T}E_{k}^{T}y\right)-e_{1}\left(e_{2}^{T}E_{i}^{T}y-e_{2}^{T}E_{k}^{T}y\right)\right)}{{\left(e_{1}^{T}E_{i}^{T}y-e_{1}^{T}E_{k}^{T}y\right)}^{2}+{\left(e_{2}^{T}E_{i}^{T}y-e_{2}^{T}E_{k}^{T}y\right)}^{2}},$$

$$u_{ik}=\frac{\left(E_{i}-E_{k}\right)(e_{3}∥E_{i}^{T}y-E_{k}^{T}y∥-\left(E_{i}^{T}y-E_{k}^{T}y\right)\left(e_{3}^{T}E_{i}^{T}y-e_{3}^{T}E_{k}^{T}y\right))}{∥E_{i}^{T}y-E_{k}^{T}{y∥}^{2}\sqrt{∥E_{i}^{T}y-E_{k}^{T}{y∥}^{2}-{\left(e_{3}^{T}E_{i}^{T}y-e_{3}^{T}E_{k}^{T}y\right)}^{2}}},$$

and $\rho ={\left[0_{1\times 3M}\;,1\right]}^{T}$, $E_{i}=\left[e_{3i-2},\;e_{3i-1},\;e_{3i}\right]$, where $e_{i}$ represents the *i*-th column of the identity matrix $I_{3M+1}$, and $e_{1}={\left[1,\;0,\;0\right]}^{T}$, $e_{2}={\left[0,\;1,\;0\right]}^{T}$ and $e_{3}={\left[0,\;0,\;1\right]}^{T}$.

Therefore, the CRLB for the estimate of the target positions is computed as:

$$\mathrm{CRB}=\mathrm{trace}\left({\left[J^{-1}\left(y\right)\right]}_{1:3M,1:3M}\right),$$

where ${\left[M\right]}_{a:b,c:d}$ represents the sub-matrix of the matrix M composed of the rows *a*–*b* and the columns *c*–*d* of *M*.
